# Targeting tumor vulnerabilities associated with loss of heterozygosity

**DOI:** 10.1080/23723556.2020.1759390

**Published:** 2020-05-13

**Authors:** Veronica Rendo, Ivaylo Stoimenov, Tobias Sjöblom

**Affiliations:** a Department of Cancer Biology, Dana-Farber Cancer Institute, USA; b Science for Life Laboratory, Department of Immunology, Genetics and Pathology, Uppsala University, Uppsala, Sweden

**Keywords:** LOH, NAT2, colorectal cancer

## Abstract

We show that N-acetyltransferase 2 (*NAT2*) loss of heterozygosity can be targeted in >4% of colorectal cancers with the use of a small molecule. We identify and describe the effect of a compound that impairs the growth of colorectal tumors with slow NAT2 activity by half when compared to wild-type.

The catalog of mutated genes known to drive cancer has been extensively characterized, but only a handful of these can be targeted with available drugs. Indeed, cancer therapy continues to rely on chemotherapeutic agents that exert their effect with limited specificity for tumor cells over normal cells in the body. Finding novel avenues for the treatment of cancers, therefore, remains a clinical priority, as most therapies show minor patient survival benefits.

A fundamental phenotype of cancer cells is their genomic instability, acquired during disease progression. Through an event known as loss of heterozygosity (LOH), alleles originally present in all of an individual’s cells are lost in the tumor counterpart, resulting in a cancer-specific reduction of genetic variation. Genomic losses through LOH are commonly selected events in the cancer genome^1^ but are not limited to regions containing tumor suppressor genes and rather include genes located in close vicinity. These bystander genes have traditionally been regarded as therapeutically irrelevant, but recent studies suggest that their involvement in housekeeping and metabolic pathways may expose cancer cells to novel therapeutic vulnerabilities. An example is collateral lethality approaches, where functional paralogues of a lost enzyme are silenced.^2-4^ Partial losses of members of the proteasome and spliceosome complexes have also been suggested as putative targets,^5^ and a current preprint study proposes the targeting of tumors that undergo LOH in essential genes.^6^ While all these studies share the concept of targeting non-driver genes in cancer, they rely on short hairpin RNA (shRNA) or Clustered Regularly Interspaced Short Palindromic Repeats (CRISPR) reagents that currently present limited therapeutic application. In our study, we investigated whether tumor allelic losses arising from LOH events can be exploited for cancer therapy with the use of small molecules.^7^

To identify putative targets for a LOH-based therapy, we mapped common variants in the 1000 Genomes project corresponding to 1,092 individuals from 14 different human populations. We focused on single nucleotide variants (SNVs) that: 1) had an allele frequency of at least 0.5%, 2) were located near catalytic residues or substrate binding pockets of the encoded protein, 3) had >5% heterozygosity frequency in all human populations, 4) were in genetic *loci* with >15% LOH frequency in at least one human cancer type, and 5) were located in genes expressed in the tumors of interest. From the resulting list of 17 putative targets, we chose to validate the N-acetyltransferase 2 (NAT2) enzyme as a candidate in colorectal cancer (CRC), as >20% patients lose a gene allele due to LOH.^8^ The NAT2 enzyme catalyzes the metabolism of xenobiotics through acetylation, and its expression is restricted to the liver and gastrointestinal tract. The identified *NAT2* SNV rs1799930 defines the NAT2*6 group of alleles, encoding slow acetylator variants with 10-fold reduced activity compared to the wild-type (NAT2*4) rapid acetylator enzyme.^9^ We envisioned that we could target a population of colorectal tumors originally heterozygous for a rapid and a slow acetylator allele, losing the rapid acetylator allele due to LOH during disease progression. These tumors would be sensitive to toxic substrates of NAT2, whereas normal colon epithelial cells and the liver maintain enzymatic activity and therefore evade toxicity (Figure 1).

**Figure 1.**
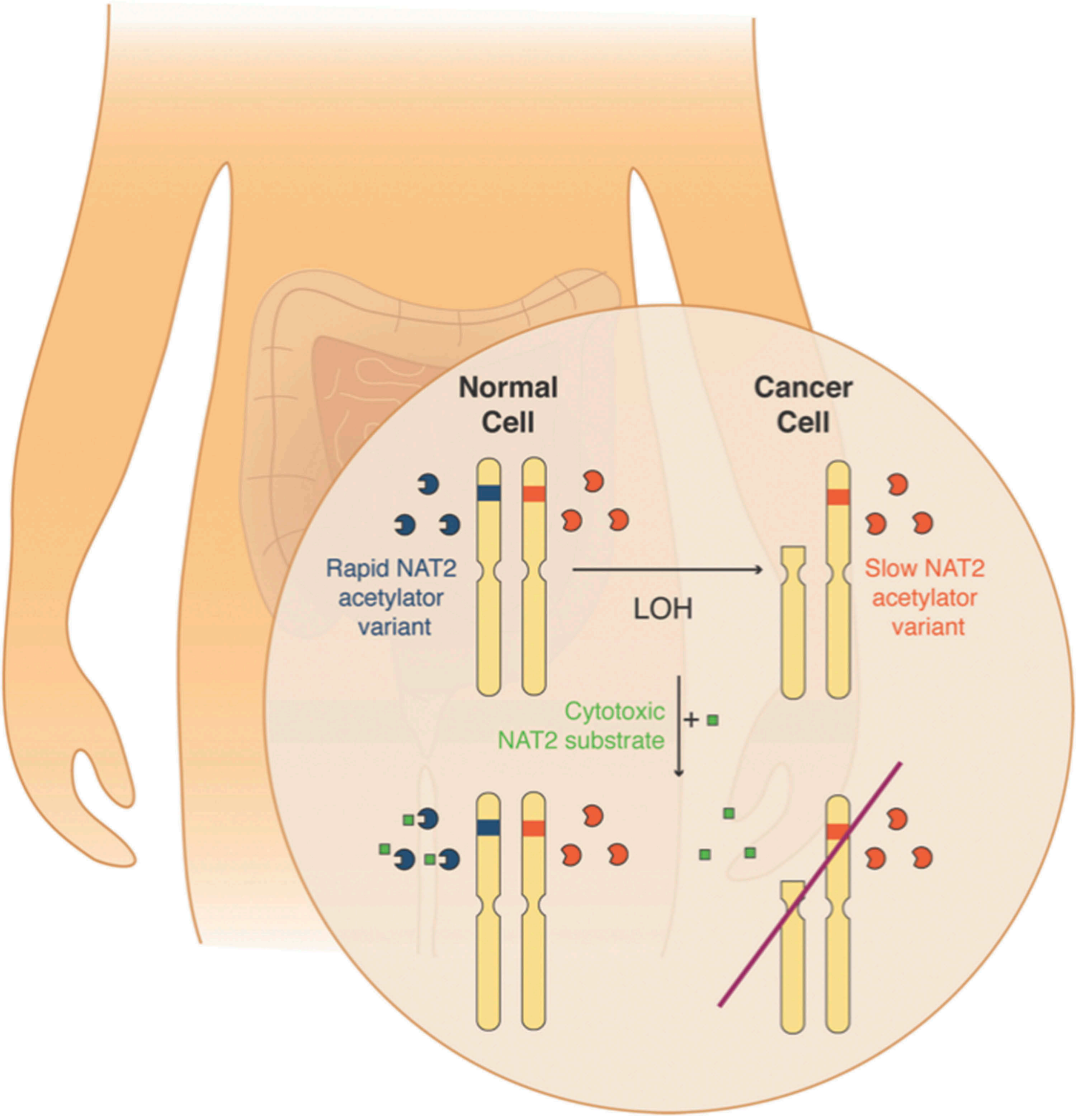
Targeting NAT2 loss of heterozygosity for cancer therapy. Eligible patients are heterozygous for the slow and rapid acetylator N-acetyltransferase 2 (*NAT2*) alleles. During disease progression, cancer cells undergo loss of heterozygosity (LOH) and lose the rapid *NAT2* allele. Treatment with a cytotoxic substrate of NAT2 will be only processed by normal cells expressing the rapid NAT2 enzymatic variant and will result in tumor-selective death. This figure was designed by V.R. and published in the Nature Research Cancer Community as part of a contribution article (https://go.nature.com/2w1uiMX).

To identify compounds that selectively kill colorectal tumors with slow NAT2, we genetically engineered the human CRC cells RKO and DLD-1 to express the rapid or slow NAT2 enzymatic variants. Detection of protein expression and confirmation of catalytic activity in the generated clones allowed us to use these cell models as tools for drug discovery. We conducted a phenotypic drug screen using a library of 176 potential NAT2 substrates, filtered by chemical structure. This effort identified the molecule 6-(4-aminophenyl)-N-(3,4,5-trimethoxyphenyl)pyrazin-2-amine (APA) as selectively toxic toward cells with slow NAT2 activity. Validation of the hit compound *in vitro* was performed through full-dose response curves in RKO and DLD-1 cells expressing rapid and slow NAT2 acetylator variants and revealed a ~threefold difference in growth inhibition between rapid and slow NAT2 cells. Structural similarities between APA and other kinase inhibitors motivated us to perform a kinome screen as a way to identify potential mediators of APA toxicity in slow NAT2 cells. We identified Aurora Kinase A (AURKA) as a putative target of APA and showed that cells with low NAT2 activity have >40% reduction in phosphorylated AURKA, become arrested at mitosis, and exhibit multiple mitotic spindles. We additionally assessed catalytic activity in our cell system, as well as binding specificity of APA to human NAT recombinant proteins. Together, we proved that rapid NAT2 cells have a~40-fold increased specificity for APA when compared to slow NAT2 cells and showed that APA acetylation is specifically mediated by NAT2 and not the human isoform NAT1.

To evaluate APA’s anti-tumor activity *in vivo*, we xenografted athymic mice on each flank with rapid and slow NAT2 tumors and delivered a liposomal formulation of APA systemically. Slow NAT2 tumors grew 50% slower than tumors expressing wild-type NAT2. Next, we evaluated the response of patient-derived primary tumor samples to APA treatment and found that tumors homozygous for slow *NAT2* acetylator alleles were, in general, more sensitive than those bearing a rapid acetylator allele.

Taken together, our study^7^ has identified allelic loss of *NAT2* as an event that can be targeted in colorectal cancers with the use of a small molecule. It further suggests that the therapeutic window of targeted therapies can be affected by the presence of bystander mutations in drug metabolic genes.
